# Factors affecting default among pre-ART patients in Eastern Uttar Pradesh

**DOI:** 10.1186/1471-2334-12-S1-P54

**Published:** 2012-05-04

**Authors:** Sangeeta Kansal, Shyam Sundar, Madhukar Rai, Narendra Kumar Tiwary, Jaya Chakravarty

**Affiliations:** 1 Department of Community Medicine and General Medicine, Institute of Medical Sciences, Banaras Hindu University, Varanasi, UP, India

## Background

Retention of patients in HIV care is a challenge as they need continuous monitoring to prevent development of advanced disease. Unfortunately even after 8 years of rolling out of ART program, there is no data on pre-ART follow up. Hence this study was conducted to identify the factors leading to default during the pre-ART period.

## Methods

This cross sectional study was conducted at ART centre, COE, BHU. All patients 18 years of age and above defaulted from pre-ART HIV care were included in the study. Defaulters were defined as any patients who missed their last appointment of CD4 count by more than one month (missed) & more than three months (lost to follow up). All these patients were traced telephonically and interviewed after taking consent. Statistical analysis was done by using SPSS version 15.0.

## Results

Out of the 1532 patients registered in pre-ART care 367 were defaulters and144 could be traced. Only 83 patients gave their consent for the interview, 73 were LFU and 10 were in the missed category. Default was common among females & patients with rural background. The main reasons for defaulting from pre-ART care were feeling of wellness 65.1%, and distance of health facility 61%.

## Conclusion and recommendations

The study recommends that there should be regular updating of contact information.

Counselling at ART centre should focus on importance of CD4 testing & its frequency.

There is a strong need to start tracking of pre-ART patients enrolled in HIV care.

